# Gut–Brain Interaction Disorders and Anorexia Nervosa: Psychopathological Asset, Disgust, and Gastrointestinal Symptoms

**DOI:** 10.3390/nu15112501

**Published:** 2023-05-27

**Authors:** Luna Carpinelli, Giulia Savarese, Biagio Pascale, Walter Donato Milano, Paola Iovino

**Affiliations:** 1 Department of Medicine, Surgery and Dentistry “Scuola Medica Salernitana”, Baronissi Campus, University of Salerno, 84081 Baronissi, Italy; lcarpinelli@unisa.it (L.C.); gsavarese@unisa.it (G.S.); b.pascale4@studenti.unisa.it (B.P.); 2 Asl Napoli2Nord, 80027 Naples, Italy; walterdonato.milano@aslnapoli2nord.it

**Keywords:** anorexia nervosa, DGBI, ROME IV criteria, body distortion, disgust

## Abstract

Background: Gastrointestinal (GI) symptoms are very common in subjects with eating disorders (EDs). This study aimed to (a) investigate the prevalence of gut–brain interaction disorders (DGBIs) in anorexia nervosa (AN) patients, according to ROME IV criteria; and (b) explore AN psychopathological assets and disgust that might impact GI symptoms. Methods: Thirty-eight female patients consecutively diagnosed with untreated AN (age 19.32 ± 5.59) in an outpatient clinic devoted to EDs underwent Eating Disorder Inventory—3 (EDI-3), Hospital Anxiety and Depression Scale (HADS), Social Phobia Anxiety Scale (SPAS), Body Uneasiness Test (BUT), and Disgust Scale (DS) questionnaires. The presence of DGBIs was evaluated and GI symptoms were assessed using a standardized intensity–frequency questionnaire. Results: A total of 94.7% of our sample met the diagnostic criteria for functional dyspepsia (FD), of which 88.8% presented the postprandial distress syndrome (PDS) subtype and 41.6% presented the epigastric pain syndrome (EPS) subtype. In addition, 52.6% of the sample met the diagnostic criteria for irritable bowel syndrome (IBS), while for functional constipation (FC), prevalence reached 7.9%. All participants presented a pathological score on the disgust scale. Significant correlations were found between several GI symptoms and psychopathological asset and disgust. Conclusions: AN is a multifactorial disorder. It is necessary to implement studies with an integrated approach, taking into account DGBIs, as well as to monitor the emotional–cognitive structure that acts as a factor in maintaining the disorder.

## 1. Introduction

Eating disorders (EDs) represent a problem of growing importance within public health and in the scientific and media spheres because of their increasingly early onset and their complex multifactorial etiology [1]. EDs are classified using the diagnostic criteria of the fifth edition of the *Diagnostic and Statistical Manual of Mental Disorders* (DSM-5), organized by the American Psychiatric Association [2]. The most frequent diagnostic categories of EDs are anorexia nervosa (AN), bulimia nervosa (BN), and binge eating disorders (BED).

Specifically, AN is a highly debilitating psychiatric disorder that impairs physical health and impairs psychosocial functioning, and it is defined by three criteria proposed by the DSM-5 [2] (see Supplementary Table S1).

In order to introduce the epidemiology of eating disorders and, particularly regarding anorexia nervosa, it should be pointed out that there are a number of methodological problems due to rejection, stigma, and shame on the part of the patient who does not always visit a doctor. These factors make such studies on the general population expensive and often ineffective, leading to a probable underestimation of the occurrence of eating disorders in the community [3].

The prevalence of EDs is increasing globally. The point prevalence estimates for AN is 0.8% [4]. The incidence rate of AN is approximately 7 cases per 100,000 people [5] and the female-to-male ratio has been found to be 13:1 [6]. In women, the highest incidence rate of AN has been shown to be around 15 years of age [7]. The incidence is increasing in Western countries due to the use of broader diagnostic criteria, earlier onset, and increased awareness [3].

### Gastrointestinal Disturbances in AN

Gastrointestinal symptoms are very common in subjects with an ED [8,9,10,11]. The mechanisms underlying this link are only partially understood and are worthy of further investigation. It has been shown that typical attitudes in EDs, e.g., dietary restriction and laxative abuse, influence gastrointestinal function, whereas altered GI function may favor typical symptoms of AN, such as reduced appetite, self-induced vomiting, and constipation [9].

There is growing knowledge on the relevance of symptoms of gut–brain interaction disorders (DGBIs) in individuals with EDs. DGBIs, formerly known as functional gastrointestinal disorders (FGIDs), are among the most common GI diagnoses worldwide [9]; they represent a heterogeneous group of disorders characterized by chronic and/or recurrent GI symptoms in the absence of structural disease. DGBIs are classified according to the ROME IV criteria [12] (see Supplementary Table S2).

A recent literature review by Hanel et al. [13] assessed how different types of DGBIs interfere with ED patients, and it was shown that patients admitted for AN frequently reported gastrointestinal symptoms, such as postprandial fullness and abdominal distension, followed by abdominal pain and early satiety. Of these patients, 40% had consulted a gastroenterologist in the period before admission [14].

In the phenotypic manifestation of such disorders, psychosocial factors such as stress and social support act as modulators of the individual patient’s experience and behavior, contributing to the clinical expression of the specific DGBI [12]. Psychopathological factors implicated in the pathogenesis of DGBI include high levels of somatization, neuroticism, anxiety [15], and depression [16].

In particular, Boyd et al. [15] showed that 98% of ED patients, 44% of whom had AN, met the ROME II criteria for at least one DGBI, and the most prevalent was irritable bowel Syndrome (IBS).

IBS is a disorder of the gut–brain axis characterized by abdominal pain and bowel disturbances in the absence of biochemical and structural causes [15], and it is frequently associated with social impairment [17]. In a study of 85 outpatients with AN, 93% reported defecation disorders of the IBS-C type (constipation variant), and prevalence increased according to both a lower body mass index (BMI) and a disease duration of more than 5 years [18].

Other scientific evidence has demonstrated the prevalence of functional dyspepsia and, in particular, postprandial distress syndrome (PDS) in 90% of AN patients and patients with other specified feeding and eating disorder (OSFED) attending an outpatient eating disorder clinic [19].

Furthermore, it has been found that once gastrointestinal symptoms develop in patients with an ED, several psycho-pathological factors interact and reinforce each other, leading to the possible persistence of functional dyspepsia (FD) independently of the pathogenesis of the ED [20].

Below, we report the diagnostic criteria for functional dyspepsia, irritable bowel syndrome, functional diarrhea, and functional Constipation [14,21] (see Supplementary Table S3).

Considering that DGBIs and AN are multifactorial disorders that possibly share several pathogenetic mechanisms, and noting the high prevalence of GI symptoms in AN evidenced through the former ROME III criteria, our scientific contribution aims to (a) investigate the prevalence of functional dyspepsia and its subgroups, namely irritable bowel syndrome and its subgroups, functional diarrhea, and functional constipation in the anorexic population according to the ROME IV criteria; and (b) explore the psycho-pathological aspects of AN that might have an impact on the symptoms reported by patients with DGBIs.

## 2. Materials and Methods

### 2.1. Procedure

Patients with AN who participated in this research study were consecutively recruited from an outpatient clinic specializing in eating disorders in ASL NA2 (Southern Italy).

The following inclusion criteria were envisaged:

- female sex,
- a recent diagnosis of AN with a severity greater than 50 calculated by EDI-3 [22,23],
- patients undergoing a complete physical examination during the first in-person visit and further investigations when indicated for the exclusion of any organic disease.

AN patients were contacted individually by telephone for an online interview appointment, which was chosen because of the restrictions, even in outpatient settings, due to the COVID-19 pandemic. The Google Meet link and informed consent form were sent by e-mail; for underage patients, one of the parents was contacted in the same way, who authorized the interview.

The interview protocol was based on 2 specific sections.

- Section A—psychopathological asset: the clinical aspects of AN were assessed by means of EDI-3, body dysmorphism were measured by means of BUT-A, disgust was measured by means of DISGUST SCALE, and the anxiety–depressive axis, also related to social phobia, were measured by means of HADS and SPAS, respectively.
- Section B—upper and lower gastrointestinal symptoms were assessed using a standardized questionnaire and the diagnoses of functional dyspepsia, irritable bowel syndrome, functional constipation, and functional diarrhea were performed according to the ROME IV Criteria.

The interviews lasted 50’–60’ and were conducted between April 2021 and January 2023. The gastroenterological interview was conducted at least 7 days after the psychiatric interview to allow for the discontinuation of any medications and supplements that might interfere with GI symptoms.

### 2.2. Materials

#### 2.2.1. Section A—AN Patients Underwent the Following Questionnaires

1. The Eating Disorder Inventory-3 (EDI-3) [22,23] is a standardized and validated questionnaire (Cronbach’s alpha = 0.70) [24] for the clinical assessment of psychological constructs known to be clinically relevant to eating disorders. Its 91 items are organized into 12 subscales, i.e., 3 that are specific to eating disorders and 9 that are general psychological scales but also relevant to eating disorders. The 3 eating disorder risk scales are as follows: drive for thinness (DT; 7 items); bulimia (B; 8 items); and body dissatisfaction (BD; 10 items), which assess attitudes and behaviors regarding eating, weight, and body shape. The 9 psychological scales are as follows: low self-esteem (LSE; 6 items); personal alienation (PA; 7 items), interpersonal insecurity (II; 7 items); interpersonal alienation (IA; 7 items), interoceptive deficits (ID; 9 items); emotional dysregulation (ED; 8 items); perfectionism (P; 6 items); asceticism (A; 7 items); and fear of maturity (MF; 8 items), which analyze the psychological traits associated with the development and maintenance of eating disorders. The test also provides 6 composite scores, i.e., 1 specific and 5 related supplementary constructs: eating disorder risk index (EDRC); inadequacy (IC); interpersonal problems (IPC); affective problems (APC); hypercontrol (OC); and general psychological maladjustment (GPMC).
2. The participants respond to the items on a 6-point Likert scale but are recoded as 0, 0, 1, 2, 3, and 4 instead of 0, 0, 0, 1, 2, and 3. The scale is standardized and can be hand-scored or computer-scored. For our study, we used the hand-scored mode for the scoring clinical report [22]. It is specified that item 71 in the original version is not included in any scale and was therefore also not included in the analyses in the present study. The pathological clinical reference cut-off based on a total sample (N = 839) is ≥50.
3. The Body Uneasiness Test (BUT) [25] is a questionnaire that explores body-image-related discomfort through various areas that cause body dissatisfaction, such as weight phobia, preoccupation, or avoidance and hypercontrol. It consists of 71 multiple-choice items on a six-point Likert-type scale (range: 0–5; from “never” to “always”) and is divided into two parts: BUT-A, consisting of 34 clinical items; and BUT-B, consisting of 37 items examining specific concerns about particular body parts or functions. For the purposes of the present study, BUT-A was used, whose clinical items provide a global severity index (GSI; the average rating of all 34 items constituting the BUT-A), which was used for statistical correlation. The other subscales were as follows: weight phobia (WP; 8 items); body image concerns (BIC; 9 items); avoidance (A; 6 items), compulsive self-monitoring (CSM; 5 items); and depersonalization (D; 6 items). Cronbach’s alpha values vary between 0.69 and 0.90. The clinical cut-off for body dysmorphism is a score ≥ 1.2.
4. The Disgust Scale-Revised (DS-R) [26] is used to assess the perception of disgust. The scale investigates the levels of perception of the primary emotion of disgust through 27 items, each of which is rated on a 5-point scale (score 0–4) with regard to the extent to which participants find the experience from not disgusting at all to very disgusting. A total score for general disgust sensitivity can be calculated. The DS-R demonstrated a high degree of internal consistency and adequate convergent and discriminant validity [27]. The cut-off for disgust dispersion is a total score > 26.
5. The Hospital Anxiety and Depression Scale (HADS) [28] is used for the assessment of the anxiety–depression axis. The test is divided into 2 components: the HADS-A consists of 7 items assessing anxiety symptoms while the HADS-D consists of 7 items assessing depressive symptoms. Each item is rated on a 4-point Likert scale (0–3) providing a maximum of 21 points for each subscale. A cut-off score of ≥8 points was applied for each subscale as this value showed good sensitivity and specificity for determining the presence of anxiety or depressive symptoms, respectively. The HADS is part of the standardized and internationally validated scales as demonstrated by a Cronbach’s alpha of 0.85.
6. The Social Phobia Anxiety Scale (SPAS) [29] is used for the assessment of phobic symptomatology related to social anxiety. It is a standardized test that assesses the presence of anxiety associated with social phobia. It comprises 12 questions assessed on a Likert-type scale ranging from 1 (strongly disagree) to 5 (strongly agree). The positively worded items (items 1, 2, 5, 8, and 11) are rated in reverse before summing. The total scores range from 12 to 60. A higher score indicates greater anxiety about the considered aspect. The internal consistency of the SPAS was reported to be 0.90 and the test–retest reliability at 8 weeks was 0.82 [30]. The clinical symptomatologic cut-off is a score of ≥20.

#### 2.2.2. Section B—AN Patients Underwent the Following Questionnaires

Patients underwent ROME IV questionnaires to diagnose IBS and FD and their subtypes. A standardized questionnaire was used to investigate the intensity and frequency of upper and lower gastrointestinal symptoms. In our outpatient clinic, a questionnaire is routinely used to detect the presence/absence, frequency from 0 to 3 (0 = none, 1 = ≤ 2 days/week, 2 = 3–5 days/week, 3 ≥ 5 days/week), and intensity from 0 to 3 (0 = null; 1 = mild: irrelevant to daily activities; 2 = moderate: influences daily activities without preventing them from being carried out; 3 = severe: markedly influence daily activities by preventing them from being carried out) of a range of upper and lower gastrointestinal symptoms.

Upper gastrointestinal tract symptoms, such as epigastric pain, epigastric burning, postprandial fullness, early satiety, upper abdominal bloating, visible upper abdominal distension, morning nausea, postprandial nausea, vomiting, and lower gastrointestinal tract symptoms (such as abdominal pain, the feeling of incomplete evacuation, straining during evacuation, and visible lower abdominal distension), were collected.

An intensity–frequency score from 0 to a maximum of 6 was obtained for each symptom. Stool consistency was recorded as a numerical value using the Bristol Stool Form Scale (BSFS). The daily measurement of the number of evacuations was summarized weekly [31,32].

### 2.3. Data Analysis

The normality of the distribution of continuous variables was tested using a one-sample Kolmogorov–Smirnov test. Continuous variables with normal distribution were presented as the mean (standard deviation [SD]), and non-normal variables were reported as the median (interquartile range [IQR]). When appropriate, a χ^2^ test was used to compare categorical data and analysis of variance (ANOVA) was used to compare normally distributed continuous variables. Spearman’s rank correlation (Rs) tests were used to correlate GI symptoms, psychopathological asset, and disgust. Significance was expressed at *p* < 0.05 level. SPSS for Windows (release 23.0; SPSS Inc., Chicago, IL, USA) was used for statistical analysis.

## 3. Results

### 3.1. Participants

Thirty-eight patients (mean age = 19.32; SD = 5.59) were consecutively recruited from the Outpatient Clinic for Eating Disorders “Asl Napoli 2 Nord” (Southern Italy).

The patients received a new incoming diagnosis of AN, according to the diagnostic criteria of the DSM-5 [2].

The sociodemographic/clinical results are reported in Table 1.

In particular, 2.7% of the variable “surgical interventions” refers to the adenoidectomy undergone by a patient included in our study. As for the variable “pharmacotherapy”, 10.5% of the sample were undergoing proton-pump inhibitor (PPI) therapy, such as lansoprazole and esomeprazole. AN patients on PPIs had bothersome intensity scores and frequency scores for epigastric symptoms more than twice a week (>2 severity score). Supplements that were taken by 44.7% of the sample included multivitamins and mineral salts.

### 3.2. Section A—Psychopathological Asset of AN

All patients presented the AN restrictive subtype (R) and presented an abnormal global psychological maladjustment index (GPMC > 50), which is one of the diagnostic criteria, and 92.1% fell within the eating disorder risk range (EDRC > 50). In fact, analyzing the means of the scales showed that the GPMC index is 132.82 (SD = 38.34), while EDRC is 50.66 (SD = 18.38) (Table 2).

Regarding body image, 100% of the sample exceeded the clinical cut-off (>1.2) in both the global index, BUT-GSI, representing body image distortion (BUT-GSI M = 3.10, SD = 0.85), and in its subscales. Specifically, the weight-related phobia dimension (BUT-WP) was found to be abnormal in 100% of the sample. Furthermore, body dispersion (BUT-BIC) exceeded the cut-off in 97.4% of the sample, while the avoidance scale (BUT-A) did so in 92.1%, the compulsive self-monitoring scale (BUT-CSM) did so in 89.5%, and the depersonalization scale (BUT-D) did so in 97.4% (Table 2)

The score on the scale relating to the primary emotion of disgust including the specific dimensions (i.e., food, animals, biological materials, death, and hygiene) was higher than the cut-off (>26) in 81.6% of the sample (Table 2).

The sample presented clinical levels for anxiety and social-anxiety-related phobia. Specifically, regarding the HADS-A, 78.9% of the sample fell within a severe clinical range, while 23.7% showed severe levels for HADS-D. Regarding the SPAS test, the total sample exceeded the indicated cut-off. (Table 2)

### 3.3. Section B—Gastroenterological Features

The prevalence of the sample that met the ROME IV criteria for the diagnosis of FD was 94.7%.

The prevalence of subtypes PDS and EPS are illustrated in Table 3.

The prevalence of the sample that fulfilled the ROME IV criteria for IBS was 52.6%, while for functional constipation (FC), prevalence reached 7.9%. None of our AN patients met the ROME IV criteria for functional diarrhea. The prevalence of IBS variants is described in Table 3.

An overlap between IBS and FD was found in 50% of our sample. In detail, 60% of patients diagnosed with IBS-C, 100% of those with IBS-D, and 100% of those with IBS-MIX met the PDS criteria. Furthermore, 60% of patients with IBS-C and 50% of the sample with IBS-MIX fulfilled the EPS criteria.

### 3.4. Relationship between the Psychopathological Asset and DGBI Diagnosis

#### 3.4.1. EDI-3

According to the ROME IV criteria, 36 out of 38 patients fulfilled those for FD and 20 out of 38 patients fulfilled those for IBS. In addition, 34 out of 36 (94.4%) patients with FD and 17 out of 20 (85%) patients with IBS showed an abnormal EDRC. There was a significant association between FD and an abnormal EDRC (χ^2^ = 17.371; *p* = 0.023).

Moreover, all FD patients with PDS had an EDRC score above the cut-off (*p* = 0.0001) with a significantly higher EDRC score than the AN patients who did not fulfill PDS criteria (53.59 ± 16.940 vs. 35.00 ± 19.318, ANOVA *p* = 0.021). Conversely, AN patients who met EPS criteria showed a significantly lower score than those without EPS (41.93 ± 13.703 vs. 56.35 ± 19.061, ANOVA *p* = 0.016). There was no difference in GMPC in any DGBI.

#### 3.4.2. BUT

Table 4 shows the results of the BUT-GSI score and of the other subscales in each DGBI diagnosis. However, the score of the global index, BUT-GSI, was significantly higher in AN patients meeting the criteria for FD subtype PDS than in AN patients not meeting the criteria for PDS (3.24 ± 0.78 vs. 2.33 ± 0.77, ANOVA *p* = 0.013).

#### 3.4.3. DISGUST

**Figure 1:**
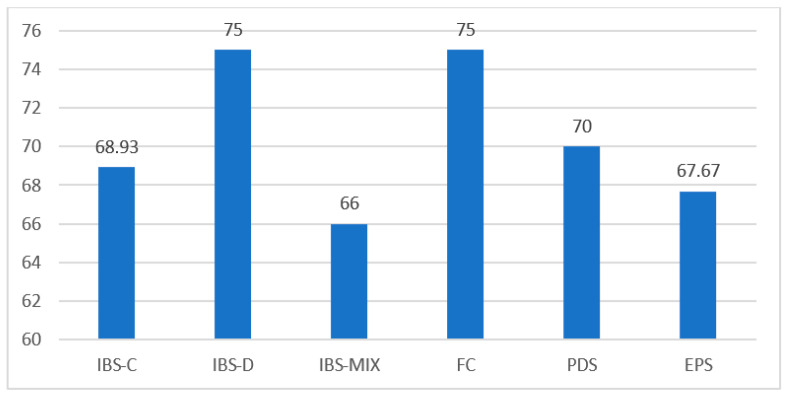
Prevalence of disgust in AN-DGBI. Irritable bowel syndrome (IBS): constipation and abdominal discomfort (IBS-C), diarrhea and abdominal discomfort (IBS-D), alternating loose stools and constipation with abdominal discomfort (IBS-mixed). Functional constipation (FC): postprandial distress syndrome (PDS), epigastric pain syndrome (EPS).

The prevalence of disgust in various DGBIs is described in Figure 1. It is not significantly associated with any DGBI. There was no significant difference in the averages of disgust scores in patients diagnosed with FD, IBS, and FC compared to AN patients who did not meet the ROME IV criteria for the above-mentioned diseases.

#### 3.4.4. HADS

A total of 14 out of 36 patients (38.8%) diagnosed with FD were classified on the basis of their HADS-D score as moderate, and 9 out of 36 patients were classified as severe (25%). For the HADS-A, 3 patients were classified as moderate (8.3%), and 28 out of 36 (77.7%) were classified as severe.

A total of 8 patients with IBS (40%) had a moderate HADS-D score, and 6 out of 20 patients (30%) were classified as severe. For the HADS-A, 17 out of 20 (85%) of the patients with IBS achieved a severe clinical range and 10% moderate were classified as moderate.

There was no significant difference in the mean HADS-D and HADS-A scores in any of the DGBIs, except for a significant increase in HADS-D in the IBS-MIX subtype.

#### 3.4.5. SPAS

All patients with FD, IBS, and FC achieved a pathological SPAS score (see Table 2). SPAS scores did not differ significantly in any DGBI.

### 3.5. Correlation of Psychological Asset and the Intensity–Frequency of Each Symptom Score

**Figure 2:**
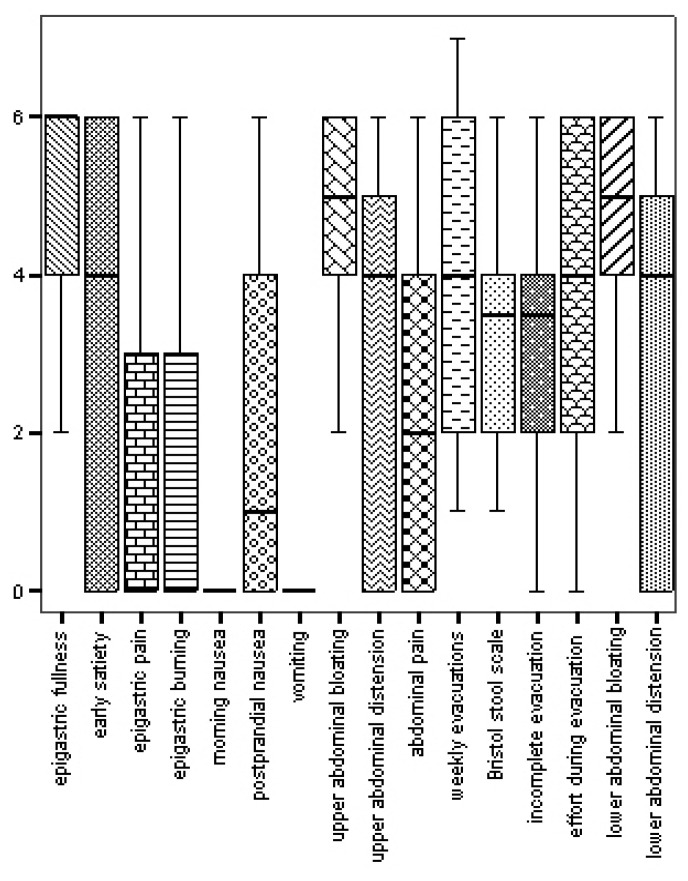
Median and interquartile range of the intensity–frequency score of upper and lower GI symptoms in AN patients.

**Figure 3:**
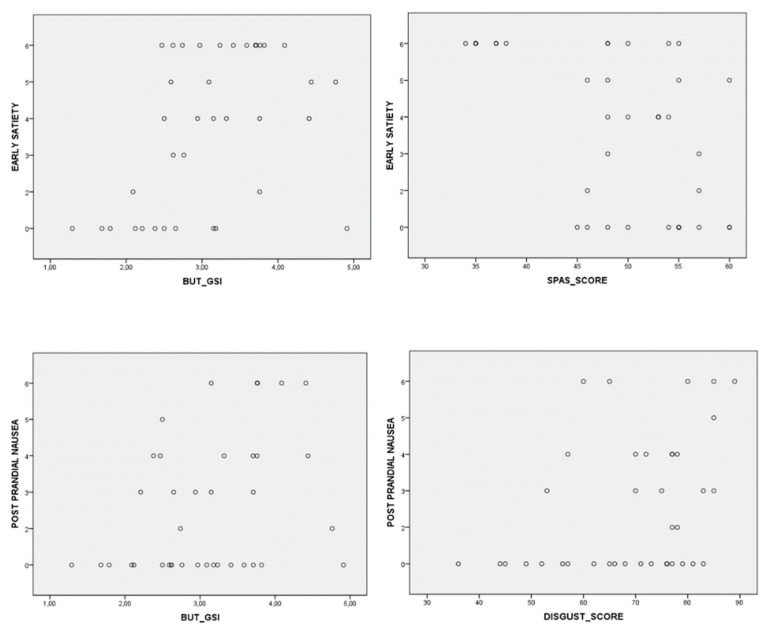
Correlation between early satiety and postprandial nausea symptoms and BUT_GSI, SPAS, and DISGUST scores.

Figure 2 shows the scores of upper and lower GI symptoms in the entire AN population. A significant correlation was found between epigastric pain and EDRC (Rs = −0.371, *p* = 0.021); postprandial nausea and BUT-GSI (Rs = 0.344, *p*= 0.034); postprandial nausea and disgust (Rs = 0.391, *p* = 0.015); early satiety and mean global severity index (GSI) of BUT (Rs = 0.462, *p* = 0.004); and early satiety and SPAS (Rs = −0.525, *p*= 0.001) (see Figure 3).

## 4. Discussion

This study demonstrated that the majority of AN patients fulfilled a diagnosis of the most common DGBIs, such as FD and IBS, according to the latest ROME IV criteria. Specifically, 94.7% of our sample met the diagnostic criteria for FD, of which 88.8% presented the PDS subtype and 41.6% presented the EPS subtype. In addition, 52.6% of the sample met the diagnostic criteria for IBS, of which 75% presented the IBS-C subtype, 5% presented the IBS-D subtype, and 20% presented the IBS-MIX subtype. No one the fulfilled ROME IV criteria for undetermined. Half of our sample fulfilled the criteria for both FD and IBS.

FD and IBS are the most common DGBIs with a prevalence ranging from 7.0% to 7.4% and from 3.9% to 4.2%, respectively, according to the ROME IV criteria [14], confirming the higher prevalence of DGBIs in AN women compared to the general population [9]. The ROME IV criteria are more stringent than the previous ROME III criteria, especially for IBS. In the current diagnostic criteria for IBS, contrary to the ROME III criteria, the term discomfort has been dropped because it has different meanings in different languages and it is an ambiguous term for patients [33].

The current definition involves a change in the frequency of abdominal pain, stating that patients must have experienced abdominal pain at least 1 day a week for the past 3 months. This contrasts with the ROME III criteria which required the presence of abdominal pain (and discomfort) at least 3 days per month [12].

In addition, the ROME III “improvement with defecation” abdominal pain parameter was changed to a “defecation-related” parameter in ROME IV because a large subset of IBS patients do not have abdominal pain improvement with defecation but report worsening. These changes in the ROME criteria might explain the lower prevalence of IBS in our sample compared to previous studies [15].

Regarding the diagnostic criteria for functional dyspepsia, the first subdivision into PDS and EPS was introduced in ROME III. In ROME IV, this distinction is broadly confirmed. The term “bothersome” was added to better describe all the symptoms present. Furthermore, in ROME III for PDS, the presence of early satiety or postprandial fullness was required at least 1 day a week versus the current 3 days a week according to ROME IV [34]. Conversely, the prevalence of FD and its variant seems unchanged compared to that reported with previous ROME criteria (ns: dyspepsia).

All participants diagnosed with any DGBI had a pathological GMPC. A total of 94.4% of patients with FD and 85% of patients with IBS had a pathological EDRC score. Interestingly, significantly higher EDRC was found in all AN patients with PDS than in the population without PDS. Conversely, the EDRC score was significantly lower.

All AN patients had a pathological BUT Global Disease Severity Score (BUT-GSI), which represents an index of total body dysmorphia. Body image is a multifactorial construct as it encompasses both the perceptual experience of one’s own body and the emotional aspect of the subject’s own body. Therefore, body image disorders support the activation of dysfunctional behaviors, such as dietary restriction and excessive weight loss, as well as influence the onset, persistence, and relapse of AN-R [35].

In our study, we demonstrated that AN patients who fulfilled the ROME IV criteria for PDS had a significantly higher BUT-GSI compared to those who did not fulfill these criteria. Moreover, a positive correlation between BUT-GSI and both early satiety and postprandial nausea were demonstrated, suggesting that body image disturbances and upper GI symptoms are possibly convergent in determining severe dietary restriction.

All participants presented a pathological score in the disgust scale, confirming the findings of previous studies [36,37]. However, we did not find any significant difference in disgust among different DGBIs. At the neural level, the DGBI patients showed activation of the anterior insula and anterior cingulate cortex [38,39,40]. The insula has been described by some authors [41,42] as the “visceral brain” due to its ability to encode interoceptive experiences that also include the gastrointestinal system. Interestingly, this neural network is also involved in disgust sensitivity [43].

In patients with AN, disgust not only has the capacity to override the biological urge to eat, drive food restriction, and vomiting [44], but it also contributes as a maintenance factor to body dysmorphism through self-repulsion. Classical aesthetic canons of beauty are widespread in today’s modern society and can be strongly internalized when they are associated with individual personality characteristics. Thus, when one’s physical appearance does not correspond to these internalized social labels concerning shape and weight, confrontation with one’s body may elicit self-loathing [35]. For these reasons, once the AN patient is in a state of hunger, biological processes may arise that further allow a vicious circle to develop, such as a decrease in appetite that could also be explained by the increase in postprandial nausea that in our study correlated significantly with disgust and BUT-GSI [42,43].

Moreover, for people with AN, their condition is not experienced as a disorder or illness to be cured, but rather as their own life choice. An “egosyntonic” phenomenon refers to a psychic condition that the subject integrates into his or her mind, making it absolutely consistent with the behavior, moods, and emotions that the person experiences every day. For this reason, vomiting is difficult to monitor in AN patients as egosyntonia does not allow them to consider it as dysfunctional behavior. Moreover, vomiting and body weight are symptoms that are not evaluated during consultations with AN patients as it could trigger anxiety states related to their current health condition. Consequently, our results on the intensity/frequency score of vomiting might have low reliability.

Furthermore, our study confirmed an important association between AN patients diagnosed with FD and anxiety–depressive symptoms. Indeed, 77.7% of this group of patients presented a severe score for the HADS-A and 25% achieved a severe score for the HADS-D. A similar finding was found in AN patients diagnosed with IBS; in fact, 85% of this sample presented a severe score for the HADS-A and 30% showed a severe score for the HADS-D. Other studies [42,43] reiterated frequent psychological comorbidities in terms of anxiety and depression in AN, FD, and IBS patients. In particular, a severe anxiety component was found in patients with IBS and FD, confirming the pivotal role of gut–brain interaction [45].

The results of the present research work suggest the importance of integrating the psychological approach with gastroenterological assessment of DGBIs, by means of screening tests for eating disorders.

However, there are several limitations in our study. One is related to sample size and another to COVID-19 restrictions. In fact, COVID-19 restrictions and lockdown measures can make gathering a large sample size for research studies challenging. Restrictions to travel, physical distancing requirements, and limited access to certain populations may result in smaller sample sizes. A smaller sample size affecting the statistical power of the study might limit the generalizability of the findings.

Furthermore, during the COVID-19 pandemic, an increase in anxiety- and depression-related symptoms was documented in EDs [46]. In fact, anxiety and depression were monitored in this study through ad hoc screening tests.

In conclusion, our study suggests the importance of a socially relevant personality trait, such as the individual disgust sensitivity and body image disturbances that act as potential triggers and/or predisposing factors for DGBI in AN patients.

It is necessary to implement studies with an integrated approach, taking into account DGBIs, as well as to monitor the emotional–cognitive structure that acts as a factor in maintaining the disorder.

## Figures and Tables

**Table 1:** Sociodemographic/clinical characteristics of AN patients (n = 38).

Variables		N (%)	Mean	SD
Age		-	19.32	5.59
BMI		-	17.63	2.23
Marital status	Single	33 (86.8)	-	-
	Cohabiting/Married	2 (5.3)	-	-
	Engaged	3 (7.9)	-	-
Smoke	Yes	5 (13.2)	-	-
	No	33 (86.8)	-	-
Alcohol	Yes	1 (2.6)	-	-
	No	37 (97.4)	-	-
Coffee	Yes	13 (34.2)	-	-
	No	25 (65.8)	-	-
Surgical intervention	Yes	1 (2.7)	-	-
	No	37 (97.3)	-	-
Pharmacotherapy	PPI	4 (10.5)	-	-
	Supplements	17 (44.7)	-	-

**Table 2:** Psychopathological asset of AN sample group.

* EDI-3 subscales **(cut-off ≥ 50)**	**Mean**	**SD**	**Min.–Max.**
EDI-DT	20.24	7.64	3–28
EDI-B	7.50	7.04	0–29
EDI-BD	22.92	7.53	9–36
EDI-LSE	14.71	5.83	3–24
EDI-PA	13.76	6.38	1–27
EDI-II	16.50	6.34	4–28
EDI-IA	12.97	5.40	4–24
EDI-ID	21.92	7.45	4–36
EDI-ED	12.97	6.76	3–28
EDI-P	9.21	4.88	0–20
EDI-A	13.18	6.07	4–28
EDI-MF	17.58	7.20	3–32
EDI-IC	28.47	11.44	4–51
EDI-IPC	29.47	10.80	10–50
EDI-APC	34.89	11.92	8–60
EDI-OC	22.39	9.31	4–48
GPMC	132.82	38.34	70–231
EDRC	50.66	18.39	16–93
** BUT subscales **(cut-off ≥ 1.2)**	**Mean**	**SD**	**Min.–Max.**
BUT-GSI	3.10	0.84	1.29–4.91
BUT-WP	3.62	1.04	1.25–5
BUT-BIC	3.25	1.02	0.89–5
BUT-A	2.39	1.06	0.17–4.67
BUT-CSM	2.58	0.90	0.67–4.17
BUT-D	3.47	1.28	1.20–6
**DISGUST Scale** **(cut-off > 26)**	**Mean**	**SD**	**Min.–Max.**
	69.26	13.12	36–89
**HADS** **(cut-off ≥ 8)**	**Mean**	**SD**	**Min.–Max.**
ANXIETY (A)	12.87	3.48	6–17
DEPRESSION (D)	7.95	3.14	4–16
**SPAS** **(cut-off ≥20)**	**Mean**	**SD**	**Min.-Max.**
	48.79	7.92	34–60

* **EDI-3**: drive for thinness (DT); bulimia (B); body dissatisfaction (BD); low self-esteem (LSE); personal alienation (PA); interpersonal insecurity (II); interpersonal alienation (IA); interoceptive deficits (ID); emotional dysregulation (ED); perfectionism (P); asceticism (A); fear of maturity (MF); inadequacy (IC); interpersonal problems (IPC); affective problems (APC); hypercontrol (OC); eating disorder risk index (EDRC); and general psychological maladjustment (GPMC). ** **BUT**: global severity index (GSI); weight phobia level score (WP); body image concerns (BIC); avoidance (A); compulsive self-monitoring (CSM); and depersonalization (D).

**Table 3:** Prevalence of functional dyspepsia (FD) and irritable bowel syndrome (IBS) subtypes in AN patients.

**Functional Dyspepsia (FD)**	**%**
PDS	88.8
EPS	41.6
**Irritable Bowel Syndrome (IBS)**	**%**
IBS-C	75
IBS-D	5
IBS-MIX	20
IBS-U	0

Functional dyspepsia (FD): postprandial distress syndrome (PDS), epigastric pain syndrome (EPS); Irritable bowel syndrome (IBS): constipation and abdominal discomfort (IBS-C), diarrhea and abdominal discomfort (IBS-D), alternating loose stools and constipation with abdominal discomfort (IBS-mixed), undefined subtype (IBS-U).

**Table 4:** Body distortion results in AN patients fulfilled a DGBI diagnosis. (M ± SD).

	IBS-C	IBS-D	IBS-MIX	FC	PDS	EPS
**BUT-GSI**	**3.04 ± 0.93**	**3.15 ± 0**	**2.98 ± 0.84**	**2.92 ± 0.82**	**3.24 ± 0.78**	**2.94 ± 0.78**
BUT-WP	3.49 ± 1.13	3.50 ± 0	3.43 ± 1.25	3.71 ± 1.25	3.74 ± 0.96	3.22 ± 1.11
BUT-BIC	3.02 ± 1.10	3.33 ± 0	3.19 ± 1.02	3.29 ± 1.22	3.38 ± 0.98	3.11 ± 0.92
BUT-A	2.62 ± 1.18	1.83 ± 0	2.04 ± 0.41	1.94 ± 1.33	2.56 ± 1.02	2.22 ± 0.99
BUT-CSM	2.51 ± 0.95	3.17 ± 0	2.66 ± 0.82	2.16 ± 0.43	2.77 ± 0.81	2.45 ± 0.76
BUT-D	3.53 ± 1.51	3.80 ± 0	3.40 ± 0.99	3.06 ± 1.02	3.59 ± 1.30	3.64 ± 1.06

**BUT**: global severity index (GSI); weight phobia level score (WP); body image concerns (BIC); avoidance (A); compulsive self-monitoring (CSM); and depersonalization (D).

## Data Availability

Written informed consent was obtained from the subject(s) in order to publish this paper.

## Supplementary Materials

The following supporting information can be downloaded at: https://www.mdpi.com/article/10.3390/nu15112501/s1, Table S1. DSM-5 criteria for AN diagnosis; Table S2. ROME IV criteria for DGBI classification; Table S3. Description of Functional Dyspepsia (FD), Irritable Bowel Syndrome (IBS), Functional Constipation (FC), and Functional Diarrhea (FD), adapted from the Fourth Edition Rome Criteria.
