# Supplementary material for: Gut–Brain Interaction Disorders and Anorexia Nervosa: Psychopathological Asset, Disgust, and Gastrointestinal Symptoms
Source: Nutrients. 2023 May 27;15(11):2501. doi: 10.3390/nu15112501 (PMC10255922; doi:10.3390/nu15112501)
Supplement: Supplementary file 1 [file nutrients-15-02501-s001.zip › nutrients-2380846-supplementary.pdf]

Table S1. DSM-5 criteria for AN diagnosis.

Anorexia Nervosa (AN)

Diagnostic criteria of DSM-5

A) Restriction of energy intake from necessary requirements such that it leads to a significantly low body weight in relation to age, gender, and developmental trajectory. Significantly low weight in adults is defined as less than the minimum normal weight (weight <18. kg/m2), in the case of children and adolescents, as less than the minimum weight expected using percentiles.

B) Intense fear of gaining weight or persistent behavior that interferes with weight gain even in the presence of an already significantly low weight.

C) Alteration in the way weight or body shape is experienced, excessive influence of weight and body shape on self-assessment or persistent lack of recognition of the severity of current body underweight.

The DSM-5 distinguishes two subtypes of AN:

1. restrictive type: during the last three months the person must not have had recurrent episodes of compulsive binge eating or emptying practices (self-induced vomiting, abuse/use of laxatives, diuretics, or enemas). This subtype describes cases in which weight loss is achieved essentially through dieting, fasting, and/or excessive exercise.

2. bulimic/purgative type: during the last three months, the anorexic person has had recurrent episodes of compulsive binge eating or elimination behavior (self-induced vomiting, abuse of laxatives or diuretics) always in the presence of clinically evident underweight.

Table S2. ROME IV criteria for DGBI classification.

**A.Esophageal Disorders**	
A1. Functional chest pain<br>*A2*. Functional heartburn<br>*A3*. Reflux hypersensitivity	*A4*. Globus<br>*A5*. Functional dysphagia
**B. Gastroduodenal Disorders**	
B1. Functional dyspepsia<br>*B1a*. Postprandial distress syndrome<br>*B1b*. Epigastric pain syndrome<br>*B2*. Belching disorders<br>*B2a*. Excessive supragastric belching<br>*B2b*. Excessive gastric belching	*B3*. Nausea and vomiting disorders<br>*B3a*. Chronic nausea-vomiting syndrome<br>*B3b*. Cyclic vomiting syndrome<br>*B3c*. Cannabinoid hyperemesis syndrome<br>*B4*. Rumination syndrome
**C. Bowel Disorders**	
C1. Irritable bowel syndrome (IBS)<br>IBS with predominant constipation<br>IBS with predominant diarrhea<br>IBS with mixed bowel habits<br>IBS unclassified<br>*C2*. Functional constipation	*C3*. Functional diarrhea<br>*C4*. Functional abdominal bloating/ distension<br>*C5*. Unspecified functional bowel disorder<br>*C6*. Opioid-induced constipation
**D. Centrally Mediated Disorders of Gastrointestinal Pain**	
D1. Centrally mediated abdominal pain syndrome	*D2*. Narcotic bowel syndrome)/ Opioid-induced GI hyperalgesia

**E. Gallbladder and Sphincter of Oddi (SO) Disorders**	
E1. Biliary pain<br>*E1a*. Functional gallbladder disorder	*E1b*. Functional biliary SO disorder<br>*E2*. Functional pancreatic SO disordes
**F. Anorectal Disorders**	
F1. Fecal incontinence<br>*F2*. Functional anorectal pain<br>*F2a*. Levator ani syndrome<br>*F2b*. Unspecified functional anorectal pain<br>*F2c*. Proctalgia fugax	*F3*. Functional defecation disorders<br>*F3a*. Inadequate defecatory propulsion<br>*F3b*. Dyssynergic defecation
**G. Childhood Functional GI Disorders: Neonate/Toddler**	
G1. Infant regurgitation<br>*G2*. Rumination syndrome<br>*G3*. Cyclic vomiting syndrome (CVS)<br>*G4*. Infant colic	*G5*. Functional diarrhea<br>*G6*. Infant dyschezia<br>*G7*. Functional constipation
**H. Childhood Functional GI Disorders: Child/Adolescent**	
H1. Functional nausea and vomiting disorders<br>*H1a*. Cyclic vomiting syndrome<br>*H1b*. Functional nausea and functional vomiting<br>*H1b1*. Functional nausea<br>*H1b2*. Functional vomiting<br>*H1c*. Rumination syndrome<br>*H1d*. Aerophagia	*H2*. Functional abdominal pain disorders<br>*H2a*. Functional dyspepsia<br>*H2a1*. Postprandial distress syndrome<br>*H2a2*. Epigastric pain syndrome<br>*H2b*. Irritable bowel syndrome<br>*H2c*. Abdominal migraine<br>*H2d*. Functional abdominal pain<br>*H3*. Functional defecation disorders<br>*H3a*. Functional constipation<br>*H3b*. Nonretentive fecal incontinence

Table S3. Description of Functional Dyspepsia (FD), Irritable Bowel Syndrome (IBS), Functional Constipation (FC), and Functional Diarrhea (FD) adapted by the Fourth Edition Rome Criteria.

**Functional Dyspepsia (FD)**
• *One or more of the following*: postprandial fullness, early satiety, epigastric pain, epigastric heartburn. All these symptoms are defined as "troublesome", severe enough to shape normal daily activities, they must have been present in the *last 3 months and occurred at least 6 months before diagnosis.*<br>• No evidence of structural disease that could explain the symptoms.<br><br>Differentiated in:<br>• **Postprandial Distress Syndrome (PDS):** at least one of postprandial fullness (severe enough to prevent you from limiting habitual activities) or early satiety (which prevents you from finishing a meal of regular quantities) at least 3 times a week.<br>Support criteria: Postprandial epigastric pain or burning, epigastric bloating, excessive belching, and nausea may be present.

- **Epigastric Pain Syndrome (EPS):** at least one of epigastric pain and/or annoying epigastric burning at least 1 day a week.

Support criteria: pain can be induced or relieved by ingestion of a meal or can occur during fasting. Postprandial epigastric bloating, nausea, and eructation may also be present.

Irritable Bowel Syndrome (IBS)

Presence of recurrent abdominal pain, at least 1 day a week, associated with 2 or more of the following parameters:

- Pain related to defecation.
- Pain associated with a change in stool frequency.
- Pain associated with a change in the shape of the stool.

The criteria must be met within the past 3 months with symptom onset at least 6 months prior to diagnosis.

Differentiated in 4 main subtypes based on the patient's reported predominant bowel habit on days with abnormal bowel movements.

The Bristol Stool Form Scale (BSFS) is used to record stool shape and consistency.

- IBS-C (constipation variant): more than a quarter (25%) of abnormal bowel movements are type 1 or 2 Bristol stools, and less than a quarter (25%) of bowel movements are type 6 or 7 Bristol stools.
- IBS-D (diarrhea variant): more than a quarter (25%) of bowel movements are Bristol type 6-7 stools and less than a quarter (25%) of bowel movements are Bristol type 1-2 stools.
- IBS-M (mixed): more than one-quarter (25%) of bowel movements are type 1-2 Bristol stools, and more than one-quarter (25%) of bowel movements are type 6-7 Bristol stools.

IBS-U (not classified): patients meet the diagnostic criteria for IBS but bowel habits cannot be accurately classified into one of the three groups above.

Functional Constipation (FC)

Must include two or more of the following:

- Straining during more than ¼ (25%) of defecations
- Lumpy or hard stools (Bristol Stool Form Scale 1-2) more than ¼ (25%) of defecations
- Sensation of incomplete evacuation more than ¼ (25%) of defecations
- Sensation of anorectal obstruction more than ¼ (25%) of defecations
- Manual maneuvers to facilitate more than ¼ (25%) of defecations (e.g., digital evacuation, support of the pelvic floor)
- Fewer than three evacuations per week
- Loose stools are rarely present without the use of laxatives.
- Insufficient criteria for IBS

The criteria must have been present in the *last 3 months and occurred at least 6 months before diagnosis.*

Functional Diarrhea

Presence of loose or watery stools, without predominant abdominal pain or bothersome bloating, occurring in more than 25% of stools.

These criteria fulfilled for the last 3 months with symptom onset at least 6 months prior to diagnosis.
Patients meeting criteria for IBS-D should be excluded.
